# Design and Preclinical Evaluation of a ^68^Ga-Labeled Macrocyclic Peptide PET Probe Targeting Transferrin Receptor 1 in Colorectal Cancer

**DOI:** 10.1021/acsomega.5c09356

**Published:** 2025-12-09

**Authors:** Yuchun Zhu, Lian Wang, Qingyan Tang, Jing Wang, Xiaoyi Zhang, Shengming Deng, Yi Yang

**Affiliations:** † Department of Nuclear Medicine, Kunshan First People’s Hospital, Kunshan 215399, China; ‡ Department of Oncology, Xuyi People’s Hospital, Huaian 211799, China; § Department of Radiology, Kunshan Jinxi People’s Hospital, Kunshan 215324, China; ∥ Department of Nuclear Medicine, Changshu No. 2 People’s Hospital, Changshu 215501, China; ⊥ Department of Nuclear Medicine, The First Affiliated Hospital of Soochow University, Suzhou 215006, China; # Nuclear Medicine Laboratory of Mianyang Central Hospital, Mianyang 621099, China; ∇ Department of Nuclear Medicine, The Second Affiliated Hospital of Soochow University, Suzhou 215002, China

## Abstract

Transferrin Receptor 1 (TfR1/CD71) is frequently overexpressed in colorectal cancer (CRC) and is associated with poor clinical outcomes, making it a compelling target for molecular imaging. However, conventional immunohistochemical (IHC) assessment is limited by spatial heterogeneity. To address this, we developed ^68^Ga-NOTA-TR01, a novel ^68^Ga-labeled macrocyclic peptide probe designed for noninvasive positron emission tomography (PET) imaging of TfR1 expression, aiming to enhance precision diagnosis and guide therapy stratification in CRC. The macrocyclic peptide precursor NOTA-TR01 was synthesized and radiolabeled with ^68^Ga. Radiochemical purity (RCP), molar activity, stability in phosphate-buffered saline and serum, and lipophilicity (log *P*) were systematically evaluated. *In vitro* characterization included surface plasmon resonance (SPR) to determine binding affinity, flow cytometry to assess TfR1 expression in CRC cell lines (HT29: high expression; LOVO: low expression), and cellular binding/blocking assays. *In vivo* performance was evaluated using microPET/CT imaging and biodistribution studies in HT29 and LOVO tumor-bearing mice, with specificity confirmed by blocking with excess unlabeled peptide. Tumoral TfR1 expression was further validated by IHC. ^68^Ga-NOTA-TR01 was synthesized with excellent RCP (>99%), high molar activity (>20 GBq/μmol), robust stability (>98% at 24 h), and hydrophilic character (log *P* = −1.709). SPR demonstrated strong binding affinity (*K*_D_ = 2.23 × 10^– 8^ M). *In vitro*, binding in TfR1^high^ HT29 cells significantly exceeded that in TfR1^low^ LOVO cells (9.56 ± 0.17% vs 4.51 ± 0.22% AD/5 × 10^5^ cells at 120 min; *P* < 0.001) and could be inhibited by over 85% with cold peptide. microPET imaging revealed specific accumulation in HT29 tumors (peak tumor-to-muscle ratio = 7.88 ± 0.79 at 120 min), which was markedly suppressed (83% reduction) by coinjection of excess unlabeled peptide. Biodistribution data confirmed significantly higher uptake in HT29 tumors (1.12 ± 0.16%ID/g vs 0.90 ± 0.21%ID/g in LOVO tumors at 120 min; *P* < 0.05), consistent with IHC results. ^68^Ga-NOTA-TR01 was a high-affinity, metabolically stable PET imaging agent capable of specifically visualizing TfR1 expression *in vivo*. This novel probe demonstrated promising potential for noninvasive patient stratification, treatment monitoring, and prognostic assessment in TfR1-positive CRC.

## Graphical abstract

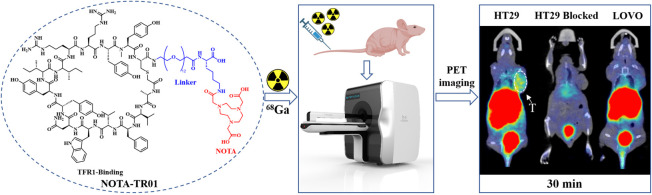

## Introduction

Over recent decades, the incidence of colorectal cancer (CRC) has risen steadily. In 2022, the global burden of CRC, including anorectal carcinomas, reached 1.93 million new cases, with approximately 904,000 related deaths, accounting for nearly 10% of worldwide cancer morbidity and mortality. CRC currently ranks third in incidence but second in cancer-related mortality.^1^ Recent predictive modeling suggests that, in the United States alone, delayed recovery of screening programs may lead to 4,000 to 7,000 excess CRC-related deaths by 2040, depending on the pace of screening resumption.^2^ These projections underscore the critical importance of early and accurate diagnosis to optimize patient outcomes.

Colonoscopy remains the clinical gold standard for CRC surveillance; however, its utility is limited by an inability to adequately characterize submucosal lesions, as well as by its invasive nature, associated patient discomfort, and contraindications in specific populations.^3^ Traditional anatomical imaging modalities, including computed tomography (CT) and magnetic resonance imaging (MRI), predominantly depend on detecting morphological alterations. This reliance renders them less effective for identifying tumors that exhibit metabolic reprogramming. In addition, these techniques intrinsically lack the capacity to elucidate the underlying molecular characteristics of tumor biology.^4^ Unlike conventional anatomical imaging modalities, such as CT and MRI, which primarily delineate structural alterations, molecular imaging enables visualization of the biological processes underlying tumor development and progression. Among molecular imaging techniques, fluorine-18 fluorodeoxyglucose positron emission tomography/computed tomography (^18^F-FDG PET/CT) has emerged as a pivotal tool for the comprehensive evaluation of CRC, encompassing initial staging, therapeutic response monitoring, and post-treatment restaging. Nevertheless, despite its diagnostic value, ^18^F-FDG PET is inherently vulnerable to false-positive uptake caused by inflammatory reactions, infectious lesions, or other benign physiological processes that mimic malignant metabolism.^5^

Transferrin receptor 1 (TfR1), also known as CD71, is a type II transmembrane glycoprotein expressed as a 180-kDa homodimer on the cell surface, where it mediates cellular iron uptake through binding to transferrin.^6^ Given iron’s essential role in DNA synthesis and cellular proliferation, TfR1 is frequently overexpressed in malignant cells. Elevated TfR1 expression has been associated with poor prognosis across multiple cancer types, including CRC. Its extracellular localization, rapid internalization kinetics, and critical involvement in tumor biology render TfR1 an appealing target for antibody-based therapies.^7–9^ Preclinical and early phase clinical evaluations of transferrin receptor 1 (TfR1)-targeted therapeutics, such as the TfR1 Probody drug conjugate CX-2029 and the anti-TfR1 antibody–drug conjugate INA03, have demonstrated potent antitumor activity, thereby substantiating the therapeutic potential of strategies centered on TfR1 modulation.^10,11^ Across a wide spectrum of targeted therapeutic modalities, clinical outcomes consistently demonstrate that tangible therapeutic efficacy is primarily realized in patients whose tumors display expression of the specific molecular determinant being targeted.^12^ For TfR1, mounting preclinical and early clinical evidence underscores that both the magnitude and spatial heterogeneity of target expression critically influence therapeutic efficacy and safety. This observation highlights the necessity of accurately quantifying TfR1 levels within tumors to guide patient selection and optimize treatment strategies.^10^ Immunohistochemistry (IHC) is widely regarded as the clinical benchmark for measuring TfR1 expression in tumor specimens. However, the variable intratumoral distribution of TfR1 often results in significant diagnostic variability. Positron emission tomography/computed tomography (PET/CT) circumvents these limitations by providing a noninvasive, quantitative, and dynamic imaging approach that captures the entire tumor’s expression profile. This strategy enables longitudinal evaluation and underpins more precise, individualized stratification of patients for TfR1-targeted therapies.^13^

Recent developments in peptide-based positron emission tomography (PET) tracers have highlighted their exceptional potential, arising from precise molecular recognition and the inherent versatility of modular design. These peptides exhibit superior tumor-targeting efficiency, enabling rapid and accurate lesion localization and supporting earlier-stage detection with enhanced diagnostic sensitivity and overall performance.^14^ Moreover, their structural adaptability allows for rational integration of chelators and radiometals, resulting in versatile probe architectures tailored to distinct biological targets and imaging needs.^15^

In this study, to enable precise diagnosis and personalized therapy for CRC, we developed a novel gallium-68 (^68^Ga)-labeled macrocyclic peptide targeting TfR1, designated ^68^Ga-TR01. The synthesized probe exhibited high binding affinity, excellent stability, and strong specificity, along with favorable pharmacokinetic properties in both *in vitro* and *in vivo* evaluations. This peptide-based PET imaging agent allows dynamic and precise visualization of TfR1 expression, offering potential to inform therapeutic decision-making and serving as a powerful modality for tumor staging, monitoring treatment response, and prognostic evaluation in clinical oncology.

## Results

### Generation and Characterization of ^68^Ga-NOTA-TR01

The synthesis of ^68^Ga-NOTA-TR01 involved the radiolabeling of TR01, a cyclic peptide composed of 15 amino acids with high affinity for TfR1 (Figure 1A). Electrospray ionization mass spectrometry (ESI-MS) analysis confirmed the expected molecular identity, with observed ion peaks at *m*/*z* 1,549.54 ([M+2H]^+^/2), 1,033.26 ([M+3H]^+^/3), 775.06 ([M+4H]^+^/4), and 620.07 ([M+5H]^+^/5), corresponding well with the theoretical mass (Figure 1B).

**Figure 1:**
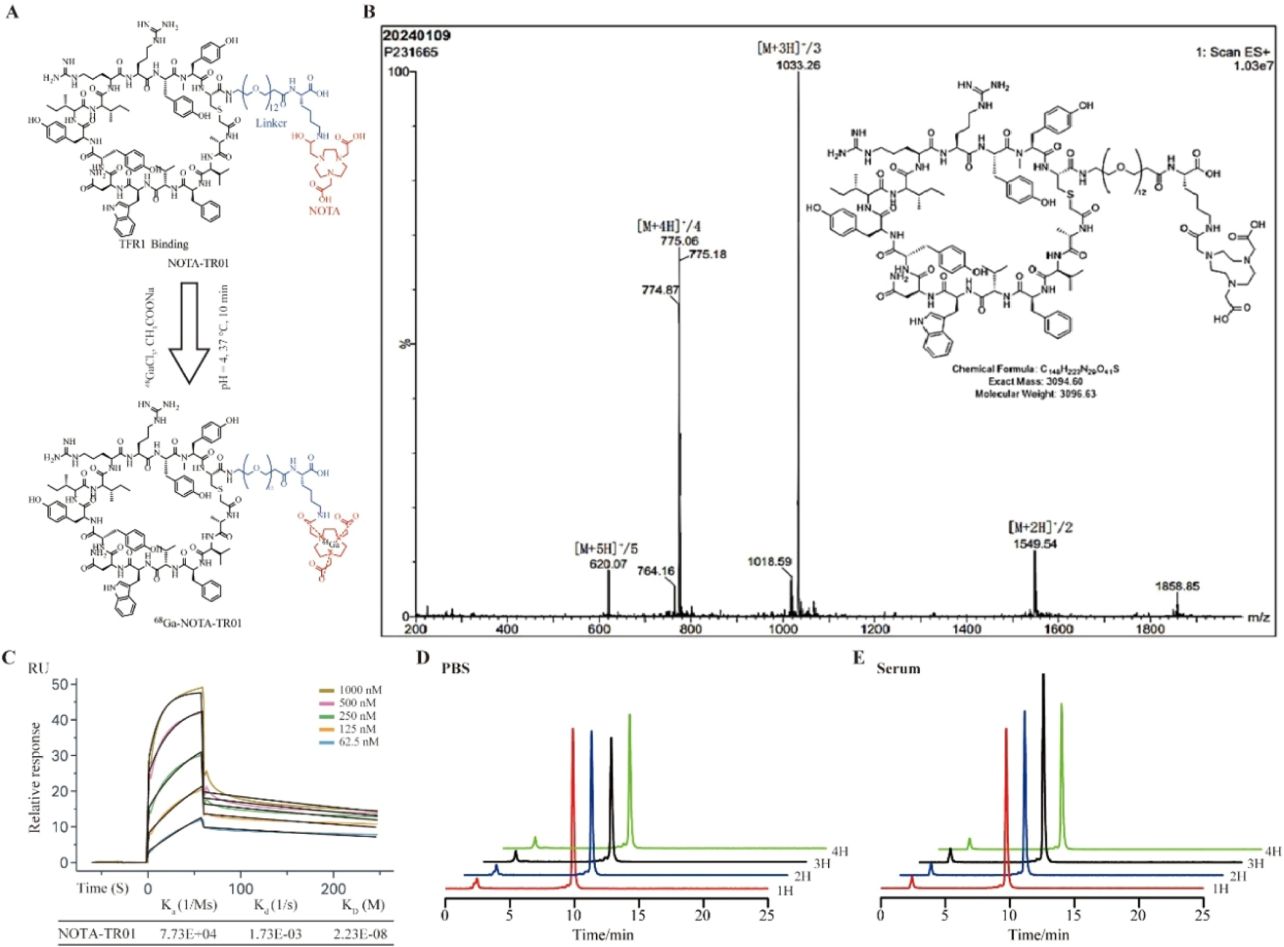
(A) Synthetic scheme for^68^ Ga-NOTA-TR01; (B) ESI-MS analysis of TR01; (C) Binding response (nm) between human TfR1 and varying concentrations (62.5–1,000 nM) of TR01; (D, E) Stability assessment of ^68^ Ga-NOTA-TR01 in PBS and serum at 37 °C, measured by radio-HPLC.

SPR was employed to quantify the binding interaction between TR01 and TfR1. As shown in Figure 1C, TR01 exhibited strong binding affinity, with a low equilibrium dissociation constant (*K*_D_ = 2.23 × 10^– 8^ M), a high association rate constant (*K*_a_ = 7.73 × 10^4^ M^– 1^ ·s^– 1^), and a low dissociation rate constant (*K*_d_ = 1.73 × 10^– 3^ s^– 1^).

The crude product of ^68^Ga-NOTA-TR01 showed an RCP exceeding 97% by radio-HPLC, which was further enhanced to >99% following purification with a C18 Sep-Pak cartridge. The molar activity was determined to be greater than 20 GBq/μmol. The log *P* was calculated as −1.709 ± 0.121 (*n* = 6), indicating a hydrophilic profile.

Stability assays demonstrated that ^68^Ga-NOTA-TR01 maintained over 98% radiochemical integrity after 24 h of incubation in PBS and human serum at 37 °C (Figure 1D,E), highlighting its excellent physicochemical stability under physiological conditions. Collectively, these findings confirmed the suitability of ^68^Ga-NOTA-TR01 for further preclinical applications.

### Cellular Binding and Target Specificity

Flow cytometry analysis was performed to evaluate TfR1 expression in LOVO and HT29 human CRC cell lines. HT29 cells showed strong surface staining with the anti-TfR1 antibody (BioLegend, Cat. No. 334103), whereas LOVO cells exhibited only weak signal intensity (Figure 2A,B), confirming differential TfR1 expression between the two lines.

**Figure 2:**
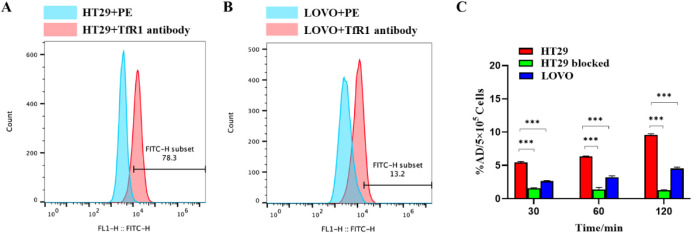
(A) Flow cytometry analysis depicting the relative expression level of TfR1 in HT29 cells. (B) Flow cytometry analysis illustrating the relative expression level of TfR1 in LOVO cells. (C) Binding of ^68^Ga-NOTA-TR01 in HT29 and LOVO cells, with or without pretreatment with 1,000-fold unlabeled NOTA-TR01, at different time points.

Following incubation at 37 °C, ^68^Ga-NOTA-TR01 exhibited significantly higher binding in TfR1^high^ HT29 cells compared to TfR1^low^ LOVO cells at all measured time points. The percentage of added dose per 5 × 10^5^ cells (%AD/5 × 10^5^ cells) was as follows: at 30 min: 5.44 ± 0.12% (HT29) vs 2.63 ± 0.09% (LOVO), at 60 min: 6.36 ± 0.05% (HT29) vs 3.19 ± 0.23% (LOVO), at 120 min: 9.56 ± 0.17% (HT29) vs 4.51 ± 0.22% (LOVO).

In the blocking group, preincubation with a 1,000-fold molar excess of unlabeled TR01 markedly suppressed ^68^Ga-NOTA-TR01 binding in HT29 cells, reducing it to 1.58 ± 0.06% at 30 min, 1.34 ± 0.29% at 60 min, and 1.27 ± 0.09% at 120 min. These results clearly demonstrated the high specificity of ^68^Ga-NOTA-TR01 for TfR1-mediated binding in tumor cells.

### PET/CT Imaging of Tumor-Bearing Mice using ^68^Ga-NOTA-TR01

Micro-PET/CT imaging was conducted in mice bearing subcutaneous HT29 or LOVO tumors to evaluate the *in vivo* distribution and tumor-targeting efficacy of ^68^Ga-NOTA-TR01 (Figure 3). Maximum intensity projection (MIP) images acquired at 30-, 60-, and 120 min postinjection revealed rapid and sustained tracer accumulation in HT29 tumors (Figure 3A,B). Quantitative analysis showed that tracer uptake in HT29 tumors reached 1.96 ± 0.24%ID/g at 30 min, decreased to 1.06 ± 0.18%ID/g at 60 min, and remained at 0.92 ± 0.18%ID/g at 120 min. Corresponding tumor-to-muscle (T/M) ratios increased over time, measuring 2.79 ± 0.36, 3.98 ± 0.81, and 7.88 ± 0.79, respectively.

**Figure 3:**
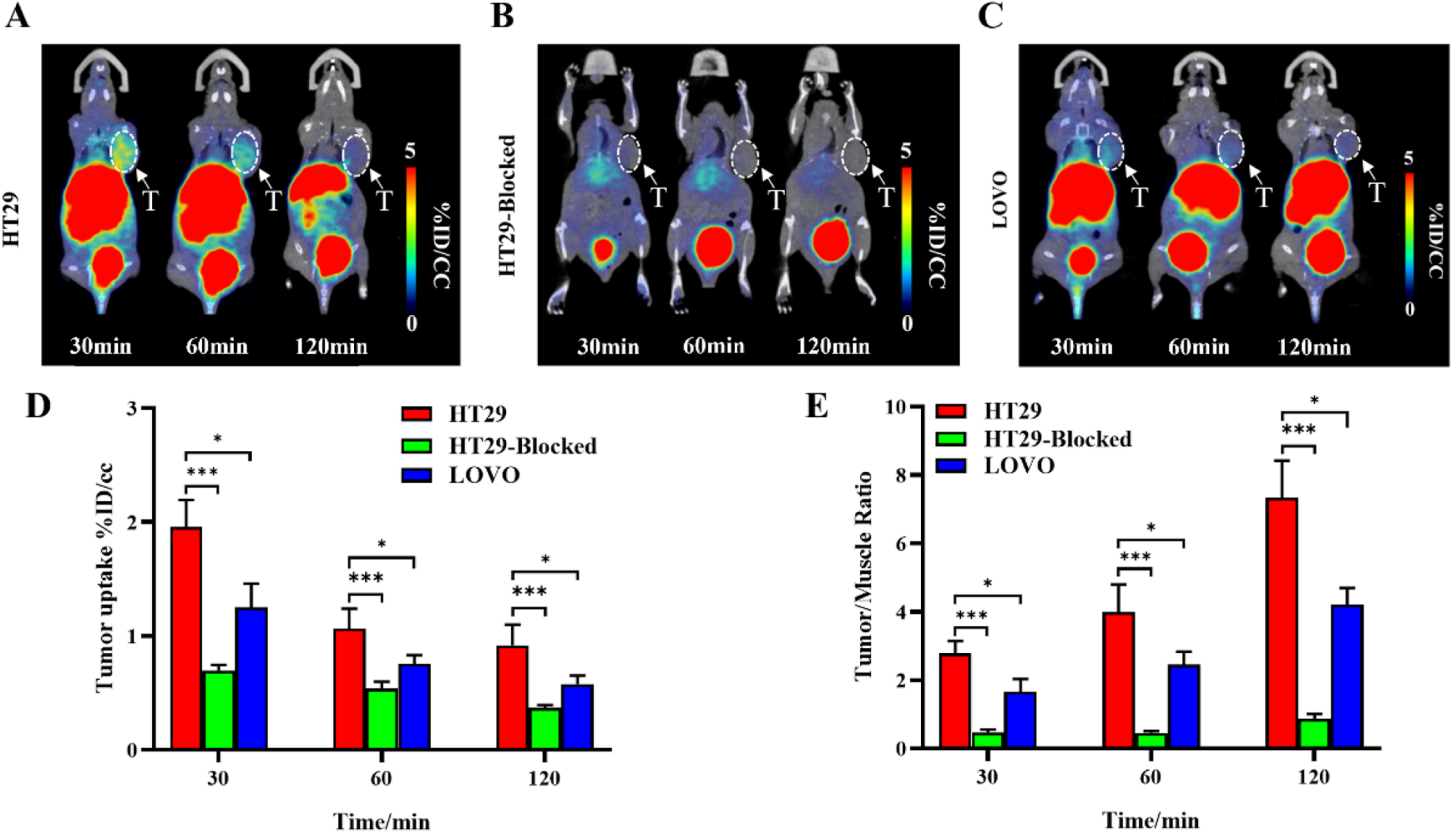
Representative PET/CT images obtained at 30, 60, and 120 min after injection of ^68^ Ga-NOTA-TR01 in HT29 tumor-bearing mice (A), LOVO tumor-bearing mice (B), and HT29 tumor-bearing mice with 1,000-fold NOTA-TR01 (C). (D, E) Tumor uptake of ^68^Ga-NOTA-TR01 and T/M ratio analyzed according to quantification analysis of PET images (*n* = 3). ***P < 0.001. *T* = tumor.

In comparison, LOVO tumors exhibited significantly lower tracer uptake at all time points: 1.25 ± 0.21%ID/g at 30 min, 0.76 ± 0.07%ID/g at 60 min, and 0.58 ± 0.07%ID/g at 120 min. The associated T/M ratios were also reduced (1.65 ± 0.38, 2.30 ± 0.40, and 2.94 ± 0.37, respectively), consistent with their lower TfR1 expression.

In blocking experiments with HT29 tumors (Figure 3C), coinjection of excess unlabeled NOTA-TR01 significantly reduced tumor uptake of ^68^Ga-NOTA-TR01 to 0.70 ± 0.05%ID/g at 30 min, 0.54 ± 0.06%ID/g at 60 min, and 0.37 ± 0.02%ID/g at 120 min. The corresponding *T*/*M* ratios decreased to 0.47 ± 0.07, 0.45 ± 0.05, and 0.86 ± 0.15, respectively, confirming the receptor-specific binding of the probe. Tumor uptake of ^68^Ga-NOTA-TR01, as well as the tumor-to-muscle (*T*/*M*) ratio measured at 30, 60, and 120 min postinjection, was significantly higher in the H29 group compared with both the LOVO group and the H29 blocking group (*P* < 0.05) (Figure 3D,E).

Notably, PET/CT images revealed prominent tracer accumulation in the kidneys and bladder, indicative of primary renal clearance. A mild hepatic signal suggested a secondary hepatobiliary excretion pathway.

### Biodistribution of ^68^Ga-NOTA-TR01 in Tumor-Bearing Mice

Biodistribution analysis in LOVO- and HT29-bearing mice (Table 1) corroborated the PET imaging findings. Uptake of ^68^Ga-NOTA-TR01 was markedly higher in HT29 tumors compared with LOVO tumors at 30 min (2.29 ± 0.14%ID/g vs 1.37 ± 0.28%ID/g), 60 min (1.20 ± 0.16%ID/g vs 1.14 ± 0.09%ID/g), and 120 min postinjection (1.12 ± 0.16%ID/g vs 0.90 ± 0.21%ID/g). Furthermore, coadministration of excess unlabeled TfR1 peptide significantly reduced tracer accumulation in HT29 tumors at 30 min (1.46 ± 0.17%ID/g vs 2.29 ± 0.14%ID/g in controls; *P* < 0.01), consistent with saturable, receptor-mediated binding.

**Table 1:** Biodistribution of ^68^Ga-NOTA-TR01 in Tumor-Bearing Mice at Different Time Points after Injection (%ID/g ; x̅±s )

	HT29			LOVO			HT29-blocked
Organ	30 min	60 min	120 min	30 min	60 min	120 min	30 min
Blood	1.68 ± 0.25	0.48 ± 0.24	0.18 ± 0.05	1.39 ± 0.37	0.36 ± 0.05	0.19 ± 0.03	2.82 ± 0.49
Tumor	2.29 ± 0.14	1.20 ± 0.16	1.12 ± 0.14	1.37 ± 0.28	1.14 ± 0.09	0.90 ± 0.21	1.46 ± 0.17
Heart	0.79 ± 0.21	0.39 ± 0.15	0.17 ± 0.04	0.63 ± 0.18	0.33 ± 0.08	0.27 ± 0.11	1.82 ± 0.63
Liver	11.70 ± 2.67	10.10 ± 1.59	13.29 ± 1.22	12.30 ± 1.32	13.98 ± 0.62	13.49 ± 1.53	8.20 ± 0.66
Spleen	2.78 ± 0.80	2.34 ± 0.25	1.37 ± 0.24	2.32 ± 0.53	2.29 ± 0.24	1.60 ± 0.16	2.38 ± 0.39
Lung	2.35 ± 0.15	1.00 ± 0.48	0.41 ± 0.07	1.20 ± 0.38	0.63 ± 0.09	0.42 ± 0.10	2.66 ± 0.51
Kidney	73.78 ± 26.20	59.34 ± 12.76	80.86 ± 15.06	59.57 ± 5.31	72.87 ± 5.86	62.80 ± 7.29	19.07 ± 3.81
Stomach	1.36 ± 0.55	0.62 ± 0.15	0.53 ± 0.29	1.01 ± 0.29	0.57 ± 0.18	0.40 ± 0.04	2.69 ± 0.68
Intestine	0.83 ± 0.21	0.43 ± 0.11	0.33 ± 0.07	0.83 ± 0.35	0.50 ± 0.06	0.55 ± 0.35	1.71 ± 0.23
Bone	0.70 ± 0.17	0.68 ± 0.14	0.43 ± 0.21	0.45 ± 0.11	0.58 ± 0.09	0.38 ± 0.10	0.95 ± 0.22
Muscle	0.61 ± 0.03	0.24 ± 0.02	0.12 ± 0.01	0.60 ± 0.07	0.36 ± 0.04	0.18 ± 0.01	0.83 ± 0.22
Brain	0.07 ± 0.02	0.03 ± 0.00	0.02 ± 0.00	0.04 ± 0.01	0.04 ± 0.01	0.02 ± 0.00	0.11 ± 0.02

Renal clearance served as the predominant excretion pathway, with peak kidney uptake observed at 30 min postinjection, accompanied by moderate hepatic accumulation. No significant tracer retention was detected in other normal tissues, including the spleen, lungs, or brain, and the low muscle radioactivity contributed to the elevated tumor-to-muscle (T/M) ratios.

In HT29 tumor-bearing mice, *T*/*M* ratios progressively increased from 3.73 ± 0.38 at 30 min, to 5.10 ± 0.34 at 60 min, and 9.18 ± 0.83 at 120 min postinjection. In contrast, corresponding *T*/*M* ratios in LOVO-bearing mice remained substantially lower at each time point, measuring 2.30 ± 0.40, 3.16 ± 0.22, and 5.01 ± 0.87, respectively.

Three mice were included at each time point; %ID/g is the percentage of injected dose per gram of tissue.

### IHC Validation of TfR1 Expression

H&E staining confirmed the histopathological characteristics of LOVO and HT29 tumor tissues. Subsequently IHC was performed to evaluate TfR1 expression and distribution (Figure 4). IHC analysis revealed strong TfR1 expression, predominantly localized to the tumor cell membranes.

**Figure 4:**
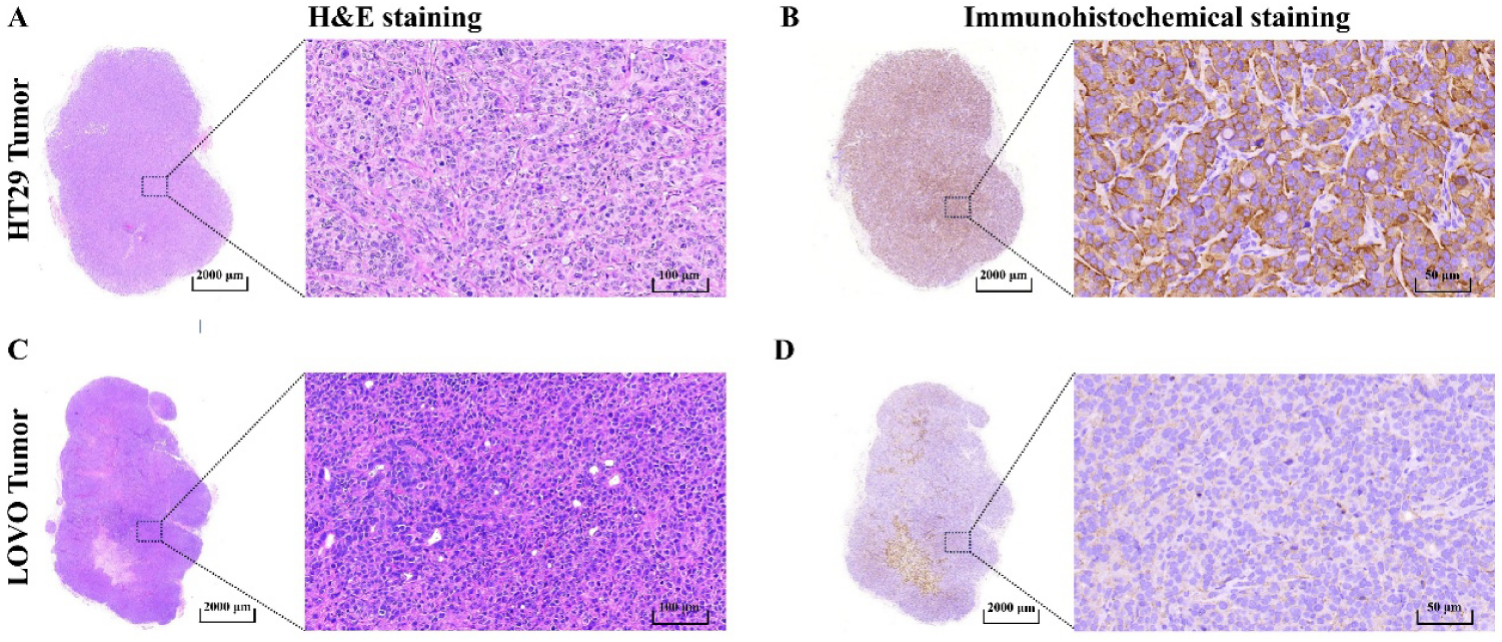
H&E staining and IHC staining of HT29 and LOVO tumor sections. H&E staining of HT29 (A) and LOVO (C) tumors. TfR1 antigen IHC staining of HT29 (B) and LOVO (D) tumors (scale bar: 2.0 mm and 100 μm).

Quantitative analysis of fluorescence intensity indicated significantly higher TfR1 expression in HT29 tumors compared with LOVO tumors. This differential expression closely mirrored the disparities in tumor uptake observed in ^68^Ga-NOTA-TR01 PET imaging, further corroborating the probe’s specificity and validating the reliability of the *in vivo* imaging results.

## Discussion

In this study, we developed and comprehensively characterized ^68^Ga-NOTA-TR01, a novel macrocyclic peptide-based PET tracer with high specificity for TfR1. The probe demonstrated excellent radiochemical purity and stability, alongside nanomolar binding affinity for TfR1. *In vitro* assays confirmed that ^68^Ga-NOTA-TR01 preferentially bound to TfR1^++^ HT29 cells, while exhibiting markedly lower affinity toward TfR1^–^ LOVO cells; this interaction was effectively competed by excess unlabeled peptide, validating its target specificity.

*In vivo* micro-PET/CT imaging and biodistribution studies further corroborated TfR1-dependent tumor uptake, with HT29 xenografts displaying pronounced tracer accumulation and high tumor-to-background contrast, whereas LOVO tumors exhibited minimal uptake. Time–activity curve analyses revealed rapid tumor delineation and sustained intratumoral retention in TfR1-positive lesions, consistent with receptor-mediated engagement. Collectively, these findings establish ^68^Ga-NOTA-TR01 as a potent molecular imaging agent for the noninvasive visualization and quantification of TfR1 expression in CRC models.

A variety of TfR1-targeted molecular imaging agents, labeled with radionuclides or fluorescent probes, have been developed, encompassing TfR1-specific monoclonal antibodies, transferrin, heavy-chain ferritin (H-ferritin, HFn), and nanoprobes.^16–21^ Despite demonstrating promising targeting capabilities, these agents are hindered by several limitations, including complex synthesis, prolonged systemic retention, increased off-target accumulation leading to elevated radiation exposure, and uncertain long-term toxicological profiles, particularly for nanomaterials.^22,23^ In this context, peptides, especially cyclic peptides, have gained prominence as molecular probes due to their superior enzymatic stability, high binding specificity, favorable pharmacokinetics, and low toxicity.^24^

To date, research on peptide-based imaging agents targeting TfR1 remains limited. A previous study has successfully identified TfR1-specific peptides on cancer cells via phage display; these peptides bind a site distinct from endogenous transferrin, thus avoiding competitive inhibition and exhibiting cellular internalization. However, their affinity was relatively modest, with dissociation constants in the micromolar range (Y1: *K*_d_ = 7.5 ± 1.0 μM; Y2: *K*_d_ = 5.3 ± 1.2 μM), and imaging studies are not conducted in that work.^25^

In contrast, we developed a novel macrocyclic peptide molecular probe, ^68^Ga-NOTA-TR01, with significantly enhanced binding affinity to TfR1 (*K*_D_ = 2.23 × 10^– 8^ M). The radiolabeled probe demonstrated high radiochemical yield, purity, and molar activity, alongside excellent stability after 4 h incubation in serum and PBS.

*In vitro* assays demonstrated robust cellular binding of ^68^Ga-NOTA-TR01 to TfR1^high^ HT29 cells, whereas binding was markedly attenuated in TfR1^low^ LOVO cells or upon competitive blocking, confirming the probe’s high affinity and specificity. *In vivo* PET imaging and biodistribution studies further validated its ability to selectively detect TfR1 expression. In HT29 tumor-bearing mice, PET scans enabled rapid visualization of TfR1-positive tumors within 30 min postinjection, with substantially higher tracer accumulation relative to LOVO tumors. Preadministration of excess nonradioactive TR01 significantly reduced tumor uptake, corroborating the *in vivo* specificity of ^68^ Ga-NOTA-TR01 for TfR1.

Biodistribution analysis revealed predominant renal clearance with minor hepatic involvement and minimal nonspecific accumulation in normal tissues such as the spleen, intestine, and lung. The tracer achieved a high target-to-background ratio, exceeding 7 at 2 h postinjection, underscoring its potential as a promising molecular probe for noninvasive visualization of TfR1 expression *in vivo*.

This study has several limitations that warrant consideration. First, imaging signals in the LOVO tumor model were relatively low, reflecting its minimal TfR1 expression. While this limits tumor detectability, it simultaneously reinforces the probe’s target specificity. Similar target-dependent imaging patterns have been reported for other ^68^Ga-labeled tracers, in which tracer accumulation closely correlates with target expression levels across different tumor types.^26^ These findings indicate that TfR1-targeted PET primarily delineates molecular target distribution rather than serving as a general-purpose tumor detection modality. Second, the probe exhibited pronounced renal uptake, raising potential concerns regarding kidney radiation exposure in clinical applications. Comparable renal retention has been observed for other ^68^Ga-labeled peptides and nanobodies, highlighting the importance of thorough dosimetric evaluation. Strategies to mitigate renal accumulation—including peptide sequence optimization, modification of linkers or net charge to reduce tubular reabsorption, and coinjection of cationic amino acids to competitively inhibit renal uptake—merit further investigation.^27^ Finally, relatively high hepatic and renal background activity may reduce tumor-to-background contrast in the abdominal region, potentially obscuring small or peri-organ lesions. Similar challenges have been encountered with other peptide-based PET tracers, such as ^68^Ga-NODAGA-RGD.^28^ Future refinements in imaging protocols, including optimization of scan timing, patient hydration strategies, and probe design, may enhance image contrast and diagnostic sensitivity.

## Conclusions

In this study, we successfully developed ^68^Ga-NOTA-TR01, a novel macrocyclic peptide–based PET probe exhibiting nanomolar-level affinity and high specificity for TfR1. Comprehensive *in vitro* and *in vivo* evaluations demonstrated its reliable ability to quantitatively and accurately assess TfR1 expression in CRC models. This molecular imaging agent holds considerable promise for facilitating precise patient stratification for TfR1-targeted antibody-drug conjugates, monitoring therapeutic target engagement, and guiding the optimization of individualized treatment regimens. Collectively, ^68^Ga-NOTA-TR01 represents a valuable tool for advancing more precise and personalized management strategies in CRC.

## Methods

### Reagents and Materials

All chemicals were of analytical grade and used without further purification. The TfR1-targeting macrocyclic peptide precursor, NOTA-TR01 [cyclo­(Ac-Ala-Val-Phe-Val-Trp-Asn-Tyr-Tyr-Ile-Ile-Arg-Arg-Tyr-NMe-Tyr-Cys)-PEG_12_-Lys-NOTA], was custom synthesized by A+ Peptide (Shanghai, China) at >95% purity. ^68^Ga was obtained from a ^68^Ge/^68^Ga generator (Eckert and Ziegler). All buffer solutions were treated with Chelex 100 resin (Aldrich) to remove metal contaminants. High-performance liquid chromatography (HPLC) and radio-HPLC analyses were conducted using a Waters pump system equipped with a C18 column (5 μm, 250 × 4.6 mm, Waters Symmetry), UV detector, and radiometric detector (Radiomatic 610TR, PerkinElmer). Radioactivity was measured using a 2480 Automatic Wizard γ-counter (PerkinElmer). Small-animal PET/CT imaging was performed on a PINGSENG Super Nova scanner (Shanghai, China).

### Preparation of ^68^Ga-NOTA-TR01

^68^Ga was eluted from a ^68^Ge/^68^Ga generator with 5 mL of 0.1 M HCl. For radiolabeling, NOTA-TR01 (50 μg), ^68^GaCl_3_ (1 mL), and sodium acetate buffer (1 M, 190 μL) were combined and incubated at 100 °C for 10 min. The reaction mixture was purified using a C18 Sep-Pak cartridge with 0.3 mL ethanol, followed by dilution with 5 mL saline. Radiochemical purity (RCP > 95%) was confirmed by radio-HPLC using a C18 column (flow rate: 1 mL/min) with a gradient elution of 15–47% acetonitrile (containing 0.1% trifluoroacetic acid) over 25 min. Detection was performed at 214 nm (UV) by radiometry.

### Partition Coefficient (Log *P*) Determination

The lipophilicity of ^68^Ga-NOTA-TR01 was determined via a standard octanol–water partition method. A solution of ^68^Ga-NOTA-TR01 [5 μL, 0.74 MBq in phosphate-buffered saline (PBS)] was mixed with 0.5 mL *n*-octanol and 0.495 mL PBS. The biphasic system was vortexed for 3 min and centrifuged at 4,000 rpm for 5 min to achieve phase separation. Aliquots (100 μL) from each layer were sampled, and radioactivity was measured using a γ-counter. The log P was calculated using the formula: log *P* = log (cpm_octanol/cpm_PBS).

### Binding Affinity Assay

The binding affinity of NOTA-TR01 to human TfR1 was evaluated by surface plasmon resonance (SPR) using a Biacore 8K system (Cytiva, USA) at Nanjing Genscript Probio. Recombinant human TfR1-Fc protein (TFR-H5264, Acro Biosystems) was immobilized on the sensor chip for kinetic analysis. The assay was performed under acidic conditions (pH 2.0) using a running buffer containing TfR1 at 30 μg/mL. Real-time binding data were collected at a 1:1 ligand-to-analyte ratio. The equilibrium dissociation constant (K_D_) was calculated by globally fitting the association and dissociation curves.

### Stability Assay

To evaluate *in vitro* stability, ^68^Ga-NOTA-TR01 (∼3.7 MBq, ∼0.3 nmol) was incubated with 200 μL of either human serum or PBS at 37 °C for 0.5, 1, and 2 h. At each time point, radiochemical integrity was assessed via radio-HPLC by comparing the retention time of the incubated samples with that of a nonincubated control.

### Cell Lines

The human CRC cell lines HT29 and LOVO were obtained from MeisenCTCC (Zhejiang, China). Cells were cultured in T25 or T75 flasks (Corning, USA) using F12K or McCoy’s 5A media as appropriate, each supplemented with 10% fetal bovine serum and 1% penicillin-streptomycin. All cells were maintained at 37 °C in a humidified atmosphere containing 5% CO_2_. Subculturing was performed using 0.25% trypsin-EDTA (Gibco, USA) upon reaching approximately 90% confluency.

### Flow Cytometry Analysis

TfR1 expression in the CRC cell lines was assessed via flow cytometry. Briefly, cells (1.0 × 10^6^ per sample) were suspended in PBS and incubated with a FITC-conjugated antihuman CD71 antibody (BioLegend, Cat. No. 334103) for 30 min at room temperature. After washing, cells were resuspended in PBS for immediate analysis. For secondary staining, cells were incubated with PE-labeled antihuman TfR1 monoclonal antibody (Enfortumab) in FACS buffer (0.5% FBS, 2 mM EDTA) for 60 min at room temperature. Following three PBS washes, a PE-conjugated antihuman IgG secondary antibody (100 μL, 1:1,000 dilution) was added. Samples were vortexed briefly and incubated in the dark for 45 min. After an additional three washes with PBS, cells were resuspended in 300 μL PBS and analyzed using a BD FACSCanto II flow cytometer. Data were processed using FlowJo v10.8 software.

### Cellular Binding Assay

The cell-specific binding of ^68^Ga-NOTA-TR01 was evaluated in HT29 and LOVO cells. Cells were seeded in 12-well plates (5 × 10^5^ cells/well) and cultured for 24 h under standard conditions (37 °C, 5% CO_2_). After washing with cold PBS, cells were incubated with 1 mL serum-free medium containing ^68^Ga-NOTA-TR01 (37 kBq, ∼3 pmol corresponding to approximately 0.4 pmol of peptide). For blocking studies, parallel wells were pretreated with a 1,000-fold molar excess (3.3 nmol, 1 μg) of unlabeled TR01 peptide. Cellular binding was assessed at 0.5, 1, 2, and 4 h postincubation. Following incubation, cells were washed twice with ice-cold PBS, lysed in 1 M NaOH, and the associated radioactivity was measured using a γ-counter. Radioactivity data were corrected for decay using the ^68^Ga decay constant (λ = 0.00876 min^– 1^). Each condition was measured in triplicate for statistical accuracy.

### Animal Studies

Xenograft tumor models were established by subcutaneously injecting 1 × 10^7^ LOVO or HT29 cells (in 100 μL PBS) into the right flank of 5–6-week-old BALB/c nude mice (purchased from Hangzhou Ziyuan Experimental Animal Technology). Tumor growth was monitored by caliper measurement, and imaging experiments were initiated once tumors reached a volume of 100–300 mm^3^. All animal procedures were conducted in accordance with the institutional guidelines of the Animal Care and Use Committee at Soochow University.

### Micro-PET/CT Imaging Protocol

Tumor-bearing mice received an intravenous injection of ^68^Ga-NOTA-TR01 (approximately 7.4 MBq, 0.6 nmol of peptide, in 150 μL). For blocking experiments, a 1 mg (0.32 μmol) dose of unlabeled NOTA-TR01 peptide was coinjected immediately prior to tracer administration. Under isoflurane anesthesia, micro-PET/CT scans were acquired at 30-, 60-, and 120 min postinjection, with each acquisition lasting 600 s (10 min). Image reconstruction and quantification were performed using PMOD software. Regions of interest (ROIs) were drawn to calculate tracer uptake, expressed as the percentage of injected dose per gram of tissue (%ID/g).

### Biodistribution Study

A comparative biodistribution study was conducted in BALB/c nude mice (*n* = 3 per group), each intravenously injected with ^68^Ga-NOTA-TR01 (1.85 ± 0.49 MBq, corresponding to ∼0.15 nmol peptide, in 100 μL saline). At 30-, 60-, and 120 min postinjection, mice were euthanized for terminal blood collection and organ harvesting. For blocking studies, an additional cohort was sacrificed at 60 min following coinjection with cold NOTA-TR01 (300 μg, ∼0.1 μmol per mouse). Excised tissues were perfused three times with PBS to remove residual blood, weighed, and their radioactivity quantified using a γ-counter. The biodistribution of the radiotracer was expressed as %ID/g. Tumor-to-background ratios (TBRs) were calculated to assess tracer specificity.

### IHC of TfR1 Expression

Tumor tissues derived from HT29 and LOVO xenografts were fixed in formalin, embedded in paraffin, and sectioned into 5-μm-thick slices. Sections were deparaffinized in xylene and rehydrated through a graded alcohol series. For hematoxylin and eosin (H&E) staining, tissue sections were stained sequentially and mounted with coverslips for histological examination. For IHC analysis, slides were incubated with goat serum at room temperature for 20 min to block nonspecific binding. Sections were then incubated overnight at 4 °C with a primary anti-TfR1 antibody (Abcam, ab214039). After washing three times with PBS, slides were treated with horseradish peroxidase (HRP)-conjugated goat antirabbit IgG secondary antibody and incubated at room temperature for 60 min. Following another three PBS washes, staining was developed using 3,3′-diaminobenzidine (DAB), and nuclei were counterstained with hematoxylin to provide morphological context.

### Data Analysis

All statistical analyses were performed using GraphPad Prism version 8.0. Data were analyzed using unpaired two-tailed Student’s *t*-tests. Quantitative values are presented as mean ± standard deviation (SD), and differences were considered statistically significant at *P* < 0.05.
